# Long-Term Stability of Polymer-Coated Surface Transverse Wave Sensors for the Detection of Organic Solvent Vapors

**DOI:** 10.3390/s17112529

**Published:** 2017-11-03

**Authors:** Ullrich Stahl, Achim Voigt, Marian Dirschka, Nicole Barié, Christiane Richter, Ansgar Waldbaur, Friederike J. Gruhl, Bastian E. Rapp, Michael Rapp, Kerstin Länge

**Affiliations:** 1 Institute of Microstructure Technology, Karlsruhe Institute of Technology, Hermann-von-Helmholtz-Platz 1, 76344 Eggenstein-Leopoldshafen, Germany; ustahl@uce.edu.ec (U.S.); achim.voigt@kit.edu (A.V.); marian.dirschka@kit.edu (M.D.); nicole.barie@kit.edu (N.B.); christiane.richter@kit.edu (C.R.); a.waldbaur@gmail.com (A.W.); friederike.gruhl@kit.edu (F.J.G.), bastian.rapp@kit.edu (B.E.R.); michael.rapp@kit.edu (M.R.); 2 Faculty of Chemical Engineering, Central University of Ecuador, Calle Ritter s/n y Bolivia, 170521 Quito, Ecuador

**Keywords:** surface acoustic wave, surface transverse wave, gas sensor, sensor array, electronic nose, volatile organic compounds, organic solvents, vapor, long-term stability

## Abstract

Arrays with polymer-coated acoustic sensors, such as surface acoustic wave (SAW) and surface transverse wave (STW) sensors, have successfully been applied for a variety of gas sensing applications. However, the stability of the sensors’ polymer coatings over a longer period of use has hardly been investigated. We used an array of eight STW resonator sensors coated with different polymers. This sensor array was used at semi-annual intervals for a three-year period to detect organic solvent vapors of three different chemical classes: a halogenated hydrocarbon (chloroform), an aliphatic hydrocarbon (octane), and an aromatic hydrocarbon (xylene). The sensor signals were evaluated with regard to absolute signal shifts and normalized signal shifts leading to signal patterns characteristic of the respective solvent vapors. No significant time-related changes of sensor signals or signal patterns were observed, i.e., the polymer coatings kept their performance during the course of the study. Therefore, the polymer-coated STW sensors proved to be robust devices which can be used for detecting organic solvent vapors both qualitatively and quantitatively for several years.

## 1. Introduction

Today, a large variety of acoustic wave devices is available, exploiting a number of different piezoelectric substrate materials and designs. These devices offer a wide range of methods for fast, sensitive, and low-cost detection of gaseous compounds. The common operation principle of acoustic sensors is based on the piezoelectric and the inverse piezoelectric effect, allowing the interconversion and detection of mechanical waves and electrical energies. The velocity of the generated acoustic waves changes linearly to changes in mass and elasticity [1,2]. Acoustic wave devices include bulk acoustic wave (BAW) devices, such as quartz crystal microbalances (QCM) using thickness shear modes (TSM), and surface acoustic wave (SAW) devices. As SAW sensors allow the use of high frequencies in the range of several 100 MHz to GHz, they allow higher mass sensitivities compared to QCMs, whose resonance frequencies are typically below 100 MHz. SAW devices use Rayleigh waves, a wave type characterized by displacement perpendicular to the surface, as well as horizontally polarized shear waves (HPSW), such as Love waves (LW) and surface transverse waves (STW). To date, the most widely accepted SAW devices for gas sensing applications have used Rayleigh waves, which were the first wave type exploited for SAW sensors [1,2,3,4,5,6].

The interdigital transducers (IDTs), i.e., the electrodes used to excite and receive the SAW on the piezoelectric material, are mainly designed in two configurations: delay line and resonator. In the delay line configuration, the input (exciting) and output (receiving) IDTs are set further apart than in the resonator configuration. The gap between the IDTs leads to a time delay between input and output signals. SAW delay line devices are typically evaluated by detection of phase and amplitude shift, which requires a complex electronic setup. However, these devices are less susceptible to manufacturing tolerances than SAW resonators. In the resonator configuration, the IDT area, defined by input and output IDTs, is surrounded on both sides (in direction of SAW propagation) by reflective structures. These reflectors act as a mass grating which keeps the SAW on the substrate surface, resulting in STW-like surface waves. Therefore, in contrast to delay line devices, SAW resonators provide very distinct and sharp resonances. The detection of the corresponding resonance frequencies is easily achievable with simple and economical electronic setups, such as oscillators [1,3,4].

The detection of specific compounds with SAW sensors requires the coating of the acoustic devices with corresponding recognition layers. Applications of SAW gas sensors include the detection of volatile organic compounds (VOCs), i.e., organic compounds which evaporate easily at ambient conditions. A multitude of coating materials for VOC detection have been developed, ranging from inorganic materials (e.g., metal oxides, carbon nanotubes), to organic molecular and supramolecular structures (e.g., self-assembled monolayers, calixarenes, cyclodextrins), and to polymer layers (e.g., pure polymer layers, molecularly imprinted polymers (MIPs)). Despite the wide range of materials, pure and simple polymer layers were not only the first, but are still the most frequently investigated vapor recognition layers for SAW gas sensors. Based on a large variety of functional groups and structures, the polymer family itself offers a wide range of coatings. The materials are typically readily available and easy to apply, e.g., by spin or spray coating [2,7]. If the polymer coating is appropriately designed, STW resonators allow higher sensitivities than (Rayleigh) SAW resonators [8,9]. Furthermore, polymer films may act as wave-guiding layers, allowing LW with increased sensitivity [3,6].

However, the polymers are not specific to individual VOCs. Rather, they allow selective detection of groups of VOCs, depending on the functional groups and, hence, the molecular interactions involved. Selectivity can be improved by the design of MIP layers, but this significantly increases the effort of the coating process. Instead, SAW sensor arrays have been designed providing SAW sensors with carefully chosen sets of different selective polymer coatings. Such arrays represent another type of electronic nose. The application of VOC samples leads to characteristic signal patterns which can be evaluated by chemometric methods. Depending on the evaluation method, both qualitative and quantitative sample characterization is possible [1,7,10].

SAW sensor arrays with selective polymer-coated devices have successfully been applied for a variety of VOC detections [1,7]. Applications include the discrimination of closely related aromatic compounds [11] or alcohols [12], monitoring of food quality by flavor-determining VOCs [13], and both classification and concentration determination of warfare agent simulants [14] or refrigerants [15]. As the polymer layers can typically be regenerated by simple flushing with clean air, multiple usage of such sensor arrays is possible and, therefore, is made use of in most applications. Multiple usage of SAW sensor arrays would imply the use in long-term applications, which would require long-term stable polymer coatings. Some studies on the development of polymer layers included investigations of the performance of the coatings for several months [8,16,17], or even years [18]. Typically, individual sensor coatings have been studied with regard to the sensor signal. However, the stability of several sensor coatings in an array and, hence, the resulting signal pattern during a longer period has hardly been investigated.

In this study, the stability of an array of eight STW resonator sensors, each one coated with a different polymer, was investigated for a period of three years. Every six months, measurements were performed with VOC samples using the organic solvents chloroform, octane, and xylene. These solvents were chosen to represent a halogenated aliphatic compound, an alkane, and an aromatic compound, respectively. Both frequency shifts and signal patterns of the array were evaluated to determine the long-term suitability of the chosen set of polymer-coated STW sensors regarding quantitative and qualitative results in VOC sensing applications.

## 2. Materials and Methods

### 2.1. STW Device and Sensor Array

**Figure 1:**
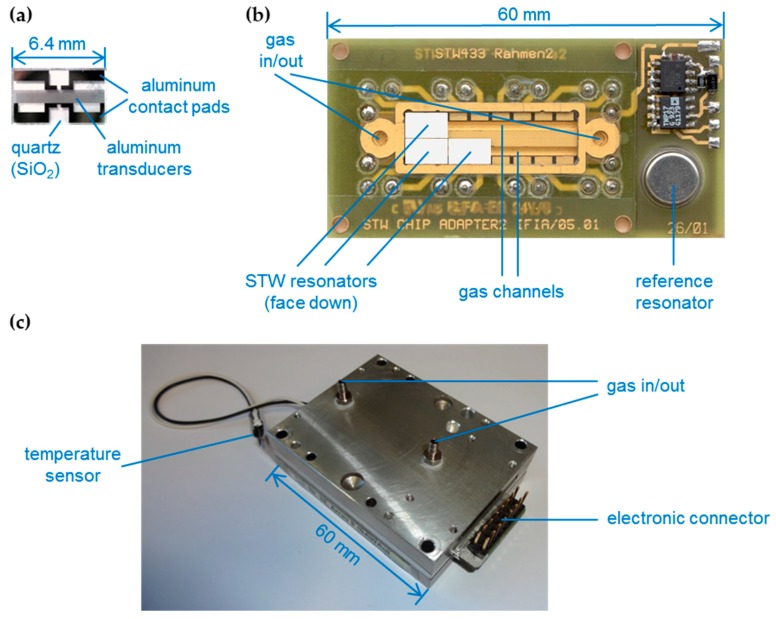
(**a**) STW resonator, *f*_0_ = 433 MHz, consisting of quartz substrate with aluminum transducers; (**b**) Sensor array with incorporated gold coated gas channels. The array is designed for eight sensors which are to be mounted face down onto the contact pads; the position of three sensors is outlined; (**c**) Metal housing with incorporated sensor array and connections to the electronics as well as to the gas flow.

STW resonators with an operating frequency of 433 MHz were supplied by SAW Components, Dresden, Germany. The resonators were based on small (6.4 mm × 3.8 mm) 36° Y-cut quartz devices (*d* = 0.5 mm) with aluminum transducers (Figure 1a).

The core element of the sensor array was an electronic board providing golden contact pads for electric coupling of the STW devices as well as gold-plated gas channels (*V* = 80 µL) for applying the gaseous samples (Figure 1b). Eight STW resonators (Figure 1a), each one coated with a different polymer (Table 1, see Section 2.2), were mounted face down onto the contact pads. By this, the devices were coupled capacitively to the driving electronics, while the active resonator structures were in contact with the gas flow. The array was integrated in a metal housing, providing electrical connections as well as connections for the gas flow (Figure 1c). An airtight cover held the devices in place and sealed the array [11,19].

Each STW resonator was integrated as frequency-determining element in an oscillator circuit developed in-house. The phase of each circuit was set by selecting an appropriate drive voltage via a capacity diode. STW resonator frequencies were determined as difference frequencies relative to a reference resonator oscillating permanently at *f*_0_^ref^ = 434 MHz while the respective phase was kept constant [20]. Therefore, processes on the sensor surface leading to frequency decrease, such as mass loading (see Table 1), led to an increase in the difference frequency. All eight difference frequencies were read out consecutively within 1 s using a multiplexing technique as described earlier [21]. The frequency resolution was 1 Hz.

### 2.2. Device Coating

The polymers used as VOC selective coatings are summarized in Table 1. Each polymer was dissolved in tetrahydrofuran (VWR, Bruchsal, Germany) at a concentration of 1 g/L. The polymer layers were applied on the STW resonators by spray-coating the respective polymer solution with an airbrush dispenser (type xy3000, purchased from Biodot, Irvine, CA, USA). The coatings were adjusted by the number of spraying steps. They were set in a way that the associated frequency shifts, which were determined by a network analyzer (type HP85046A, Hewlett-Packard, Amstelveen, Netherlands), were below 2 MHz (Table 1). Using such thin layers, the mass effect on the sensor signal prevails potential elastic effects [8,9]. The resulting sensor signals correlated linearly to the solvent vapor concentrations applied in this study (Section 2.3).

### 2.3. Samples, Measurement Setup and Measurement Protocol

Chloroform, octane, and xylene (all purchased from VWR, Bruchsal, Germany) were used as solvent vapor samples. Table 2 summarizes the solvent properties of these VOCs. To create a defined solvent vapor atmosphere a poly(methyl methacrylate) (PMMA, Plexiglas^®^) box, *V* = 130 L, equipped with a sample plate and a fan was used. Defined volumes of solvent were pipetted onto the sample plate and subsequently evaporated and homogeneously distributed by the fan. Table 3 summarizes the drop volumes and resulting vapor concentrations of the VOCs in the PMMA box.

**Figure 2:**
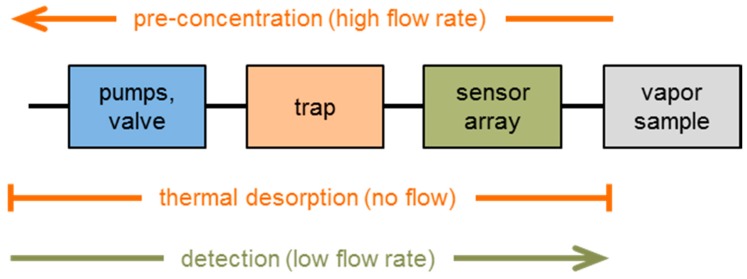
Schematic of the measurement setup with STW sensor array and pre-concentration unit (“trap”). The setup was connected to a 130 L PMMA box in which the defined solvent vapor atmosphere was generated.

The STW sensor array was connected to the vapor-filled PMMA box and to a pre-concentration unit developed in-house. This unit, called “trap” in the following, consisted of a glass tube filled with sorbent material. In this work, the filling was Tenax^®^ TA (Sigma-Aldrich, Supelco division, Taufkirchen, Germany), a porous polymer adsorbent based on 2,6-diphenylene oxide. Details and pictures of the complete measurement setup have been published earlier [11,19,22]; a schematic is shown in Figure 2.

**Figure 3:**
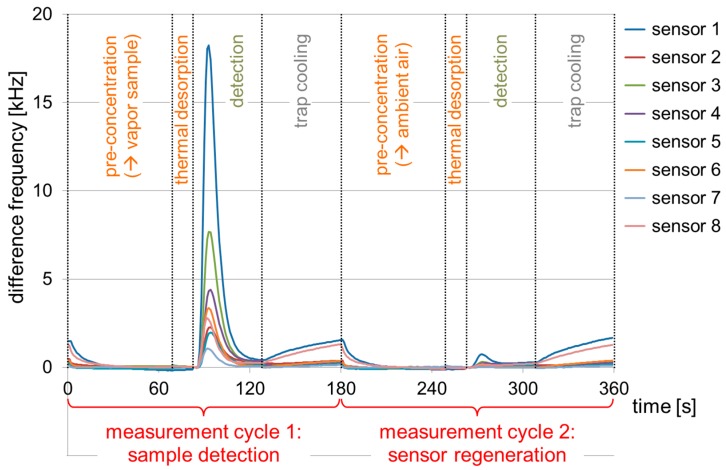
STW sensor array signal responses during measurement cycles for detection and regeneration. Chloroform, 11.6 ppmv, was used as exemplary sample. Dotted lines separate the cycle steps. Each cycle consisted of pre-concentration of gaseous sample (solvent vapor or ambient air) in the trap, thermal desorption, detection by the sensor array, and trap cooling.

A measurement cycle consisted of pre-concentration, thermal desorption, detection, and trap cooling. During the pre-concentration phase, a pump delivered the contents of the PMMA box, i.e., the solvent vapor sample, across the sensors to the trap. The uptake of solvent molecules in the sensor coatings (and, hence, the sensor signal responses) remained negligible here, because of the comparatively small vapor concentrations (Table 3); however, solvent molecules were enriched in the trap material. This was followed by the thermal desorption step, at which the trap was heated up to 200 °C to release the solvent molecules. After that, another pump delivered the concentrated vapor across the sensor array where it was detected. Afterwards, the trap was cooled down, and the sensors were regenerated by repeating the measurement cycle at ambient air. The number of regeneration cycles depended on boiling point and concentration of the solvent applied (Table 2 and Table 3). In this work, a complete measurement cycle required only 3 min (Figure 3), and a maximum of three regeneration cycles was required.

Measurements were performed every six months for a period of three years; in between, the sensor array remained in the metal housing (Figure 1c). For storage, the housing, together with the array, was removed from the measurement setup. Gas inlet and outlet were interconnected by a polytetrafluoroethylene (PTFE) tube. The sensor array remained in the sealed housing and was stored in an electronics laboratory with an average temperature of 23 ± 4 °C and a relative humidity of 55 ± 15%. Before each use, several regeneration cycles (i.e., measurement cycles at ambient air) were carried out prior to the actual experiment.

### 2.4. Data Evaluation

At the start of the detection step (Figure 3) all sensor signals, i.e., difference frequencies, were reset to zero. The sensor signal maxima obtained during the detection were determined. These difference frequency shifts correlated linearly to the solvent vapor concentrations applied (Section 2.2 and Table 3). In addition, the eight signal maxima within a detection step were divided by the corresponding maximum value, i.e., the highest of these eight signal maxima. The resulting normalized difference frequency shifts were plotted as radar charts to obtain characteristic patterns for the organic solvents which were applied as VOC samples.

## 3. Results and Discussion

### 3.1. Absolute Difference Frequency Shifts

**Figure 4:**
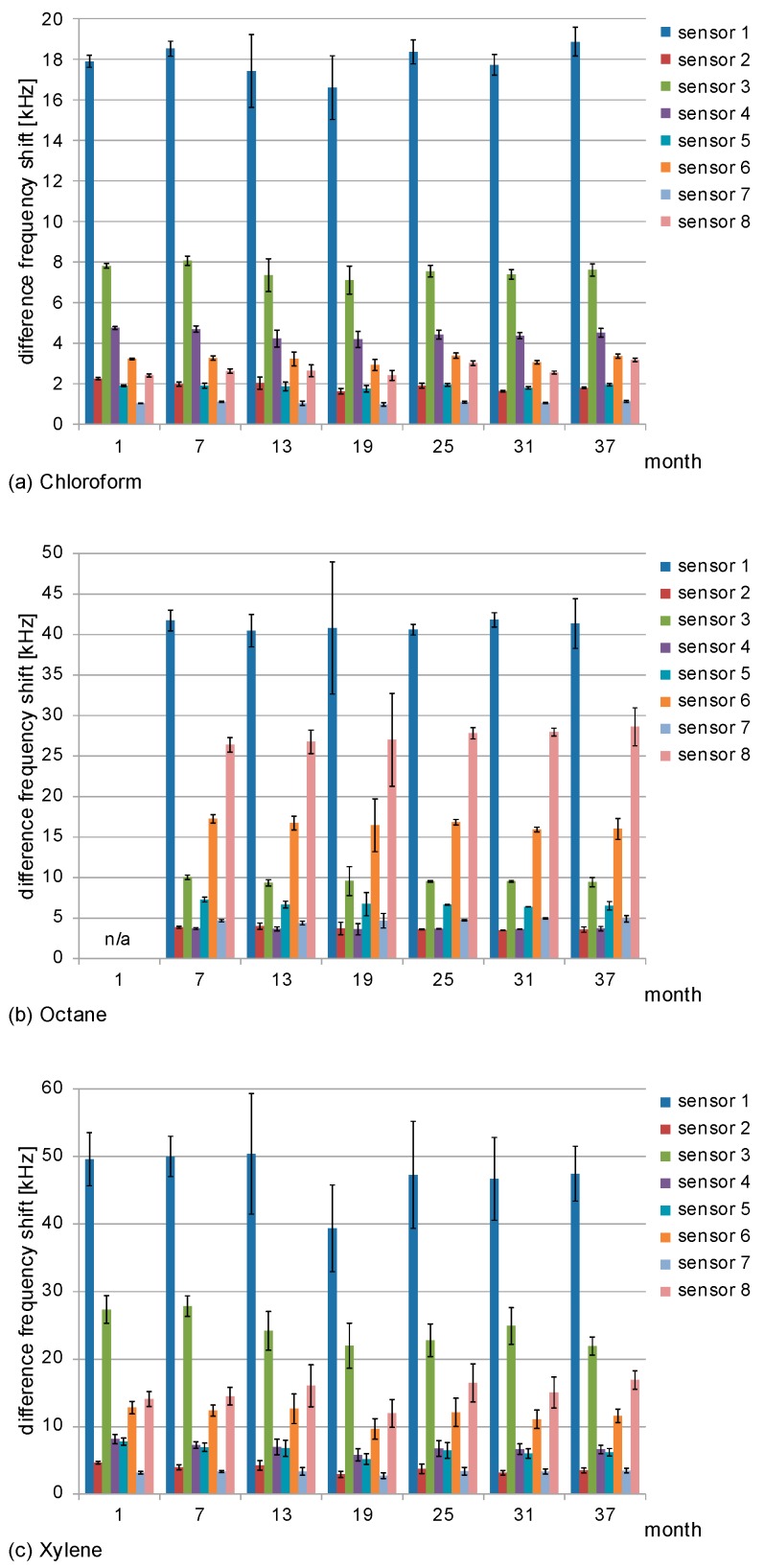
Difference frequency shifts obtained with the STW sensor array. Samples were (**a**) chloroform, 11.6 ppmv; (**b**) octane, 11.5 ppmv; (**c**) xylene, 4.5 ppmv. Measurements were performed in triplicate every six months for a period of three years with the exception of octane, which was included in the study half a year later. Columns represent the means; error bars represent the standard deviations of the measurements (*n* = 3).

For a period of three years, measurements with the STW sensor array were performed every six months. A halogenated aliphatic compound (chloroform), an alkane (octane), and an aromatic compound (xylene) were used as solvent vapor samples. Each solvent was applied in three concentrations (Table 3). The difference frequency shifts obtained during the three-year period for one exemplary concentration per solvent are summarized in Figure 4. Furthermore, mean and standard deviation values of the difference frequency shifts as well as relative standard deviation values are listed in Table S1 (Supplementary Materials).

As shown in Figure 4 and Table S1, there was no significant increase or decrease of the sensors’ difference frequency shifts within the standard deviation range which could be associated with the time course of the study. This was observed for all sensors and, hence, sensor coatings, as well as for all solvent vapor samples applied, including the samples of the other concentrations whose results are not shown here. Regarding the difference frequency shifts on the individual measurement dates, the majority (65%) of the relative standard deviations were below 10%; but partly the values ranged up to 20% or higher. However, increased relative standard deviations were not linked with later measurement dates. Instead, increased values were mainly observed when smaller solvent volumes had to be applied to obtain the desired vapor concentrations (Table 3), such as 3 µL xylene to obtain 4.5 ppmv as shown in Figure 4c. In this study, the solvents were manually pipetted into the PMMA box. Variations in the pipetting process have a stronger impact on small sample volumes than on larger ones and, hence, on the subsequent vapor concentrations and resulting sensor signals. Aside from higher standard deviations, pipetting errors may also lead to systematic errors, i.e., higher variations in consecutive measurements. Still, considering the average difference frequency shifts during the three-year period of the study, no significant increase or decrease linked with the time course could be observed. Standard deviations and systematic errors of potential further measurements can easily be reduced by an optimized sample application procedure.

Sensor 1 (PBMA coating) provided the highest difference frequency shifts for each of the solvent vapor samples. Still, as shown in Figure 4a,b, the signal response of sensor 1 to octane was higher than that to chloroform, despite the fact that the molar masses of those two solvents are similar (Table 2) and similar concentrations had been used (11.5 ppmv and 11.6 ppmv, respectively) (Table 3). The other sensors’ difference frequency shifts also varied in association with the type of the solvent sample applied. These observations confirmed that the different polymer coatings responded differently to the solvent vapors, as required for a sensor array, depending on the respective polymer’s selectivity and layer thickness. Based on these results, signal patterns could be derived which were characteristic for the corresponding solvent. Details are discussed in the next section (Section 3.2).

**Figure 5:**
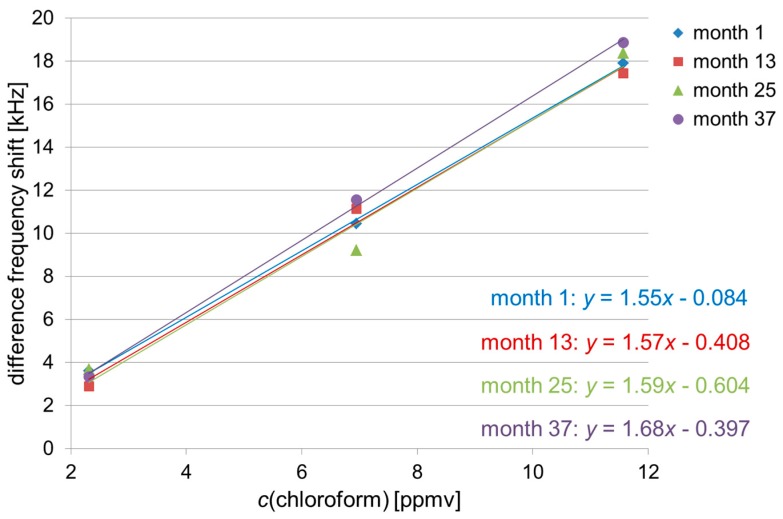
Mean values of sensor 1 (PBMA coating) obtained with chloroform samples at the concentrations of 2.3 ppmv, 6.9 ppmv, and 11.6 ppmv at annual intervals. The lowest and highest concentrations were measured in triplicates, the medium concentration in duplicates. For the sake of clarity, error bars are not included. Regression lines were calculated with *y* = difference frequency shift [kHz] and *x* = *c*(chloroform) [ppmv].

**Figure 6:**
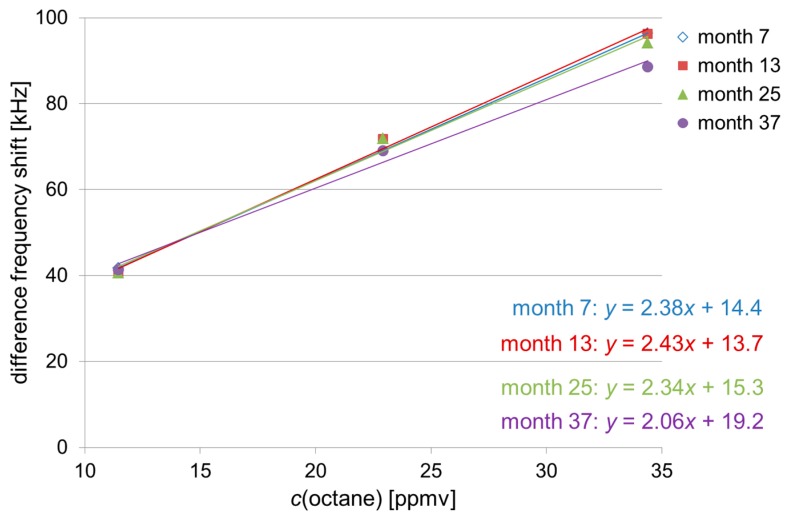
Mean values of sensor 1 (PBMA coating) obtained with octane samples at the concentrations of 11.5 ppmv, 22.9 ppmv, and 34.4 ppmv at (semi-)annual intervals: As month 1 is missing, year 1 is represented by month 7. The lowest and highest concentrations were measured in triplicate, the medium concentration in duplicate. For the sake of clarity, error bars are not included. Regression lines were calculated with *y* = difference frequency shift [kHz] and *x* = *c*(octane) [ppmv].

**Figure 7:**
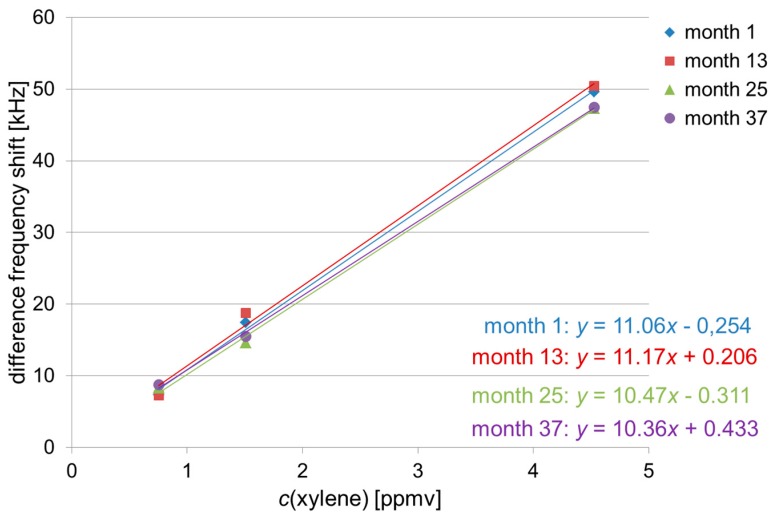
Mean values of sensor 1 (PBMA coating) obtained with xylene samples at the concentrations of 0.8 ppmv, 1.5 ppmv and 4.5 ppmv at annual intervals. The highest concentration was measured in triplicate, the others in duplicate. For the sake of clarity, error bars are not included. Regression lines were calculated with *y* = difference frequency shift [kHz] and *x* = *c*(xylene) [ppmv].

The polymer coatings were adjusted in a way that a linear correlation between difference frequency shift and corresponding solvent vapor concentration was obtained (Section 2.2 and Table 3). Hence, if a solvent vapor sample is identified by the signal pattern (Section 3.2), the absolute difference frequency shift allows the calculation of the sample concentration. In Figure 5, Figure 6 and Figure 7 the difference frequency shifts of sensor 1 are plotted versus the respective solvent vapor concentrations (Table 3), and the lines of best fit are included. Sensor 1 was chosen because, as mentioned above, it provided the highest difference frequency shifts for all solvent vapor samples (Figure 4). For the sake of clarity, only measurements at annual intervals have been included, and error bars representing standard deviation values have been omitted. As octane was included in the study half a year later than chloroform and xylene, a semi-annual interval precedes the two final annual intervals in Figure 6.

For each solvent, the respective lines of best fit obtained during the three-year period were similar. As the lines of best fit represent the calibration lines, the sensitivities (i.e., slope of the calibration line) of the array also remained similar for each solvent. Slight deviations as observed, e.g., with the latest line (month 37) of the octane measurements (Figure 6) were not significant considering the respective deviation range. As depicted above, the deviations largely resulted from errors in pipetting. Therefore, to allow a more exact quantification in the future, the sampling procedure should be optimized, e.g., by an automated sample injector.

### 3.2. Normalized Difference Frequency Shifts and Signal Patterns

**Figure 8:**
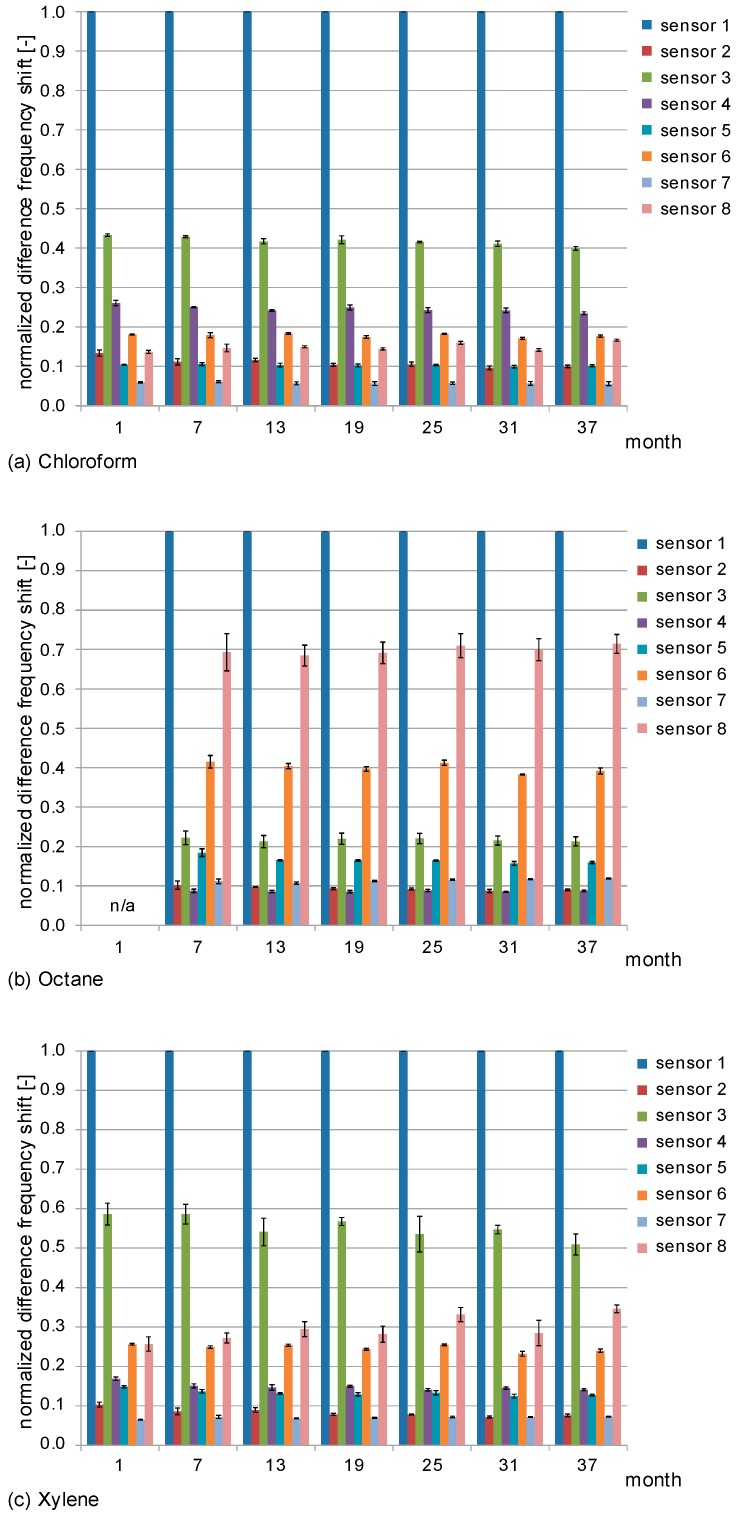
Normalized difference frequency shifts obtained with the STW sensor array. Samples were (**a**) chloroform; (**b**) octane and (**c**) xylene. One representative measurement was chosen from each of the three solvent vapor concentrations; and the difference frequency shifts of all sensors were normalized to the corresponding difference frequency shift of sensor 1 (i.e., the maximum difference frequency shift). Columns represent the means; error bars represent the standard deviations of the measurements (*n* = 3).

The polymer coatings react selectively with the solvent vapor samples. Hence, an STW array with differently coated STW sensors provides patterns of signal responses that are characteristic for the corresponding solvents. The patterns can already be guessed from the absolute difference frequency shifts as shown in Figure 4, but they become better recognizable and comparable if they are normalized to the respective highest values. Figure 8 shows the normalized difference frequency shifts obtained during the three-year period. From each of the three solvent vapor concentrations used in this study (Table 3), one representative measurement was chosen. For each of those measurements, the difference frequency shifts of all sensors were normalized to the corresponding maximum difference frequency shift, i.e., the difference frequency shift of sensor 1 (Figure 4). Furthermore, mean and standard deviation values of the normalized difference frequency shifts as well as relative standard deviation values are listed in Table S2 (Supplementary Materials).

The normalized difference frequency shifts (Figure 8 and Table S2) were similar within the standard deviation range, i.e., no significant time-related change of the normalized signals was observed. This is in accordance with the previous results obtained with the absolute difference frequency shifts (Section 3.1). The vast majority (96%) of the relative standard deviations on the individual measurement dates were below 8%; and none of the values was higher than 11%. This means that the relative standard deviations of the normalized difference frequency shifts were significantly smaller than those obtained with the absolute difference frequency shifts. This is because the normalized difference frequency shifts are independent of the sample volume; therefore, variations in the pipetting process do not interfere with the normalized sensor signals. Hence, even if the sample application method is not optimized, a qualitative analysis can still be performed.

**Figure 9:**
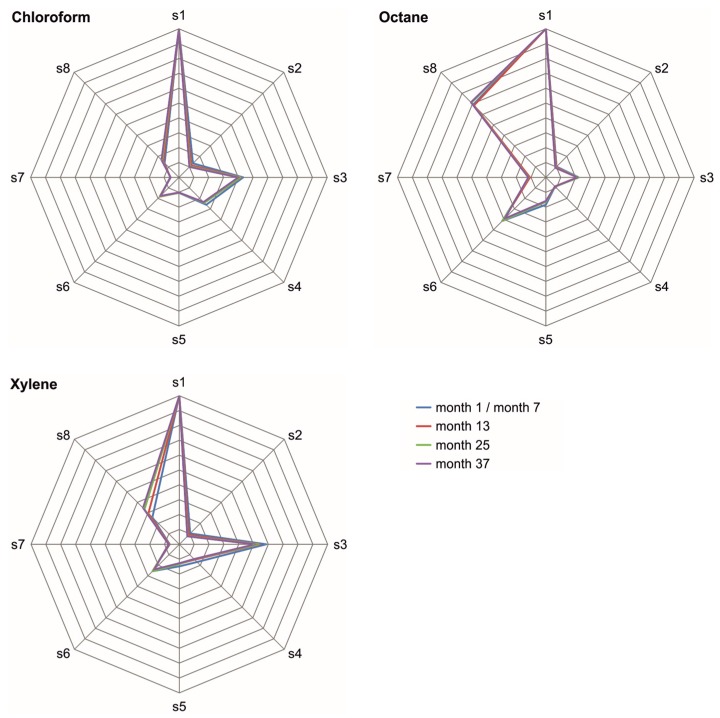
Radar charts of chloroform, octane, and xylene samples obtained at (semi-)annual intervals. Chloroform and xylene measurements started at month 1, octane measurements at month 7; s1–s8 represent sensors 1–8. One representative measurement was chosen from each of the three solvent vapor concentrations; and the difference frequency shifts of all sensors were normalized to the corresponding difference frequency shift of sensor 1 (i.e., the maximum difference frequency shift; see Figure 8). The normalized difference frequencies were averaged (*n* = 3). For the sake of clarity, error bars are not included.

Figure 9 shows the normalized difference frequency shifts displayed in Figure 8 plotted as radar charts to make the signal patterns clearly recognizable. For the sake of clarity, only measurements at annual intervals have been included, and error bars representing standard deviation values have been omitted. Characteristic patterns were obtained for each of the solvents. Slight deviations as observed, e.g., for the xylene patterns during the course of the study were not significant considering the respective standard deviation range (Figure 8). Therefore, despite the deviations, the respective patterns remained characteristic for the corresponding solvents during the complete three-year period.

## 4. Conclusions

Measurements with a gas sensor array consisting of eight STW resonator sensors coated with different polymers were performed semi-annually for a period of three years. The results obtained during the course of this study showed no significant time-related changes in the sensor signals. Consequently, the polymer-coated sensors maintained their performance, and no noticeable aging effects could be observed. It was possible, during the three years, to distinguish the patterns of chloroform as an exemplary chlorinated hydrocarbon, octane as an exemplary aliphatic hydrocarbon, and xylene as an exemplary aromatic hydrocarbon for the entire duration of the study. Furthermore, the sensitivity of the array remained the same, allowing concentration determination throughout the three years. However, to allow a more exact quantification with reduced standard deviations in the future, an automated sampling method instead of manual pipetting would be recommended.

## Figures and Tables

**Table 1:** Polymer coatings of STW resonators, *f*_0_ = 433 MHz, used as VOC sensors.

Sensor No.	Polymer	Supplier	Frequency Shift by Spray Coating [MHz]
1	PBMA: Poly(butyl methacrylate)	Aldrich, Milwaukee, WI, USA	−1.1138
2	Polyurethane alkyd resin with trace isocyanates	RS Components, Corby, UK	−0.7400
3	PECH: Polyepichlorohydrin	Aldrich, Milwaukee, WI, USA	−1.3271
4	Silar-10C	Alltech, Deerfield, IL, USA	−0.4404
5	PCFV: Poly(chlorotrifluoro ethylene-*co*-vinylidene fluoride)	Aldrich, Milwaukee, WI, USA	−1.0000
6	L grease	Apiezon, London, UK	−0.6004
7	PDMS: Polydimethylsiloxane	Macherey-Nagel, Düren, Germany	−0.4304
8	PIB: Polyisobutylene	Aldrich, Milwaukee, WI, USA	−1.1233

**Table 2:** Properties (manufacturer specifications) of the organic solvents used for the VOC sensing experiments.

Solvent	Molecular Formula	Relative Molar Mass *M_r_*	Boiling Point [°C]	Density [g/cm³]
Chloroform	CHCl_3_	119.38	61	1.48
*n*-Octane	C_8_H_18_	114.23	125–126	0.70
Xylene ^1^	C_8_H_10_	106.17	137–143	0.86–0.88

^1^ Technical mixture of o-, m-, and p-xylene.

**Table 3:** Applied volumes and concentrations in mg/m^3^ and ppmv (parts per million volume) of the organic solvents used for the VOC sensing experiments.

Solvent	Drop Volumes [µL]	Concentrations [mg/m^3^]	Concentrations [ppmv]
Chloroform	1; 3; 5	11.4; 34.2; 57.0	2.3; 6.9; 11.6
*n*-Octane	10; 20; 30	54.1; 108.2; 162.2	11.5; 22.9; 34.4
Xylene ^1^	0.5; 1; 3	3.3; 6.6; 19.8	0.8; 1.5; 4.5

^1^ Technical mixture of o-, m-, and p-xylene.

## Supplementary Materials

The following are available online at http://www.mdpi.com/1424-8220/17/11/2529/s1, Table S1: Difference frequency shifts obtained with the STW sensor array, Table S2: Normalized difference frequency shifts obtained with the STW sensor array.
