# Supplementary material for: Long-Term Stability of Polymer-Coated Surface Transverse Wave Sensors for the Detection of Organic Solvent Vapors
Source: Sensors (Basel). 2017 Nov 3;17(11):2529. doi: 10.3390/s17112529 (PMC5713134; doi:10.3390/s17112529)
Supplement: Supplementary file 1 [file sensors-17-02529-s001.zip › Sensors-232631_Supplementary-Materials_Table-S1.pdf]

Table S1. Difference frequency shifts obtained with the STW sensor array.

Samples were (**a**) chloroform, 11.6 ppmv, (**b**) octane, 11.5 ppmv, (**c**) xylene, 4.5 ppmv. Measurements were performed in triplicates every six months for a period of three years with the exception of octane, which was included in the study half a year later. The following tables summarize mean ± standard deviation (S) values of the difference frequency shifts as well as relative standard deviations (RSD) obtained for each measurement date (n = 3). RSD values in the table result from unrounded mean and standard deviation values.

Table S1. (**a**) Chloroform, 11.6 ppmv

Sen-sor No.	Month 1		Month 7		Month 13		Month 19		Month 25		Month 31		Month 37	
	Mean ± S [kHz]	RSD [%]	Mean ± S [kHz]	RSD [%]	Mean ± S [kHz]	RSD [%]	Mean ± S [kHz]	RSD [%]	Mean ± S [kHz]	RSD [%]	Mean ± S [kHz]	RSD [%]	Mean ± S [kHz]	RSD [%]
1	17.90 ± 0.30	1.7	18.52 ± 0.37	2.0	17.42 ± 1.80	10.3	16.60 ± 1.57	9.5	18.37 ± 0.59	3.2	17.72 ± 0.51	2.9	18.87 ± 0.71	3.8
2	2.25 ± 0.06	2.6	1.98 ± 0.11	5.5	2.03 ± 0.30	14.9	1.63 ± 0.14	8.4	1.91 ± 0.13	6.5	1.63 ± 0.04	2.6	1.80 ± 0.04	2.4
3	7.81 ± 0.12	1.5	8.07 ± 0.23	2.8	7.35 ± 0.80	10.9	7.11 ± 0.69	9.6	7.56 ± 0.28	3.7	7.40 ± 0.24	3.2	7.61 ± 0.31	4.0
4	4.75 ± 0.07	1.5	4.69 ± 0.16	3.3	4.22 ± 0.42	9.9	4.19 ± 0.40	9.5	4.42 ± 0.21	4.8	4.37 ± 0.15	3.5	4.51 ± 0.21	4.7
5	1.90 ± 0.04	1.9	1.91 ± 0.13	6.6	1.86 ± 0.21	11.5	1.75 ± 0.16	9.2	1.94 ± 0.07	3.6	1.80 ± 0.06	3.5	1.95 ± 0.06	2.9
6	3.22 ± 0.04	1.3	3.27 ± 0.11	3.4	3.22 ± 0.34	10.6	2.92 ± 0.27	9.3	3.39 ± 0.13	3.9	3.06 ± 0.09	2.9	3.36 ± 0.11	3.3
7	1.04 ± 0.01	0.8	1.11 ± 0.03	2.8	1.03 ± 0.11	10.6	0.98 ± 0.09	9.1	1.10 ± 0.05	4.2	1.05 ± 0.03	2.9	1.13 ± 0.04	3.6
8	2.41 ± 0.07	2.7	2.62 ± 0.10	4.0	2.64 ± 0.29	11.1	2.41 ± 0.24	10.0	3.01 ± 0.11	3.8	2.55 ± 0.08	3.0	3.17 ± 0.09	2.8

Table S1. (**b**) Octane, 11.5 ppmv

Sen-sor No.	Month 1		Month 7		Month 13		Month 19		Month 25		Month 31		Month 37	
	Mean ± S [kHz]	RSD [%]	Mean ± S [kHz]	RSD [%]	Mean ± S [kHz]	RSD [%]	Mean ± S [kHz]	RSD [%]	Mean ± S [kHz]	RSD [%]	Mean ± S [kHz]	RSD [%]	Mean ± S [kHz]	RSD [%]
1	n/a	n/a	41.73 ± 1.28	3.1	40.48 ± 2.00	4.9	40.81 ± 8.14	19.9	40.60 ± 0.65	1.6	41.81 ± 0.89	2.1	41.37 ± 3.07	7.4
2	n/a	n/a	3.86 ± 0.14	3.5	3.99 ± 0.40	10.0	3.72 ± 0.77	20.8	3.60 ± 0.05	1.4	3.47 ± 0.03	1.0	3.58 ± 0.33	9.3
3	n/a	n/a	10.04 ± 0.28	2.8	9.36 ± 0.39	4.1	9.57 ± 1.79	18.6	9.54 ± 0.10	1.0	9.55 ± 0.10	1.0	9.43 ± 0.56	5.9
4	n/a	n/a	3.70 ± 0.11	3.1	3.67 ± 0.24	6.6	3.63 ± 0.70	19.3	3.68 ± 0.05	1.3	3.61 ± 0.04	1.0	3.69 ± 0.27	7.2
5	n/a	n/a	7.29 ± 0.29	4.0	6.67 ± 0.41	6.1	6.73 ± 1.43	21.2	6.65 ± 0.07	1.0	6.40 ± 0.03	0.4	6.53 ± 0.53	8.0
6	n/a	n/a	17.24 ± 0.52	3.0	16.72 ± 0.85	5.1	16.46 ± 3.26	19.8	16.83 ± 0.34	2.0	15.93 ± 0.29	1.8	16.00 ± 1.30	8.1
7	n/a	n/a	4.69 ± 0.14	3.0	4.39 ± 0.24	5.4	4.68 ± 0.91	19.4	4.74 ± 0.08	1.7	4.95 ± 0.08	1.7	4.92 ± 0.40	8.1
8	n/a	n/a	26.39 ± 0.90	3.4	26.76 ± 1.45	5.4	26.99 ± 5.73	21.2	27.82 ± 0.70	2.5	27.96 ± 0.48	1.7	28.61 ± 2.34	8.2

Table S1. (**c**) Xylene, 4.5 ppmv

Sensor No.	Month 1		Month 7		Month 13		Month 19		Month 25		Month 31		Month 37	
	Mean ± S [kHz]	RSD [%]	Mean ± S [kHz]	RSD [%]	Mean ± S [kHz]	RSD [%]	Mean ± S [kHz]	RSD [%]	Mean ± S [kHz]	RSD [%]	Mean ± S [kHz]	RSD [%]	Mean ± S [kHz]	RSD [%]
1	49.59 ± 3.92	7.9	50.00 ± 2.98	6.0	50.40 ± 8.93	17.7	39.35 ± 6.42	16.3	47.28 ± 7.93	16.8	46.70 ± 6.14	13.1	47.45 ± 4.06	8.5
2	4.67 ± 0.21	4.4	3.99 ± 0.38	9.4	4.24 ± 0.72	17.0	2.92 ± 0.47	16.0	3.75 ± 0.70	18.7	3.17 ± 0.33	10.5	3.50 ± 0.37	10.6
3	27.35 ± 2.06	7.5	27.86 ± 1.51	5.4	24.19 ± 2.87	11.9	21.98 ± 3.33	15.2	22.80 ± 2.42	10.6	24.92 ± 2.71	10.9	21.93 ± 1.33	6.1
4	8.17 ± 0.67	8.2	7.28 ± 0.50	6.8	6.99 ± 1.15	16.4	5.81 ± 0.88	15.2	6.76 ± 1.17	17.2	6.67 ± 0.79	11.9	6.63 ± 0.64	9.6
5	7.78 ± 0.52	6.7	6.94 ± 0.63	9.0	6.79 ± 1.20	17.6	5.19 ± 0.79	15.3	6.48 ± 1.18	18.2	6.01 ± 0.70	11.6	6.21 ± 0.56	9.0
6	12.81 ± 0.92	7.2	12.37 ± 0.82	6.6	12.65 ± 2.19	17.3	9.66 ± 1.52	15.8	12.10 ± 2.10	17.4	11.09 ± 1.38	12.4	11.60 ± 1.00	8.6
7	3.18 ± 0.19	6.1	3.35 ± 0.14	4.2	3.36 ± 0.57	17.0	2.75 ± 0.43	15.6	3.40 ± 0.56	16.6	3.35 ± 0.39	11.5	3.49 ± 0.32	9.3
8	14.10 ± 1.11	7.8	14.50 ± 1.30	9.0	16.05 ± 3.10	19.3	11.96 ± 2.07	17.3	16.46 ± 2.81	17.0	15.06 ± 2.32	15.4	16.91 ± 1.38	8.2
