# Supplementary material for: Long-Term Stability of Polymer-Coated Surface Transverse Wave Sensors for the Detection of Organic Solvent Vapors
Source: Sensors (Basel). 2017 Nov 3;17(11):2529. doi: 10.3390/s17112529 (PMC5713134; doi:10.3390/s17112529)
Supplement: Supplementary file 1 [file sensors-17-02529-s001.zip › Sensors-232631_Supplementary-Materials_Table-S2.pdf]

Table S2. Normalized difference frequency shifts obtained with the STW sensor array.

Samples were (**a**) chloroform, (**b**) octane, and (**c**) xylene. One representative measurement was chosen from each of the three solvent vapor concentrations (see Table 3); and the difference frequency shifts of all sensors were normalized to the corresponding difference frequency shift of sensor 1 (i.e., the maximum difference frequency shift). The following tables summarize mean ± standard deviation (S) values of the difference frequency shifts as well as relative standard deviations (RSD) obtained for each measurement date (n = 3). RSD values in the table result from unrounded mean and standard deviation values.

Table S2. (**a**) Chloroform

Sensor No.	Month 1		Month 7		Month 13		Month 19		Month 25		Month 31		Month 37	
	Mean ± S [-]	RSD [%]	Mean ± S [-]	RSD [%]	Mean ± S [-]	RSD [%]	Mean ± S [-]	RSD [%]	Mean ± S [-]	RSD [%]	Mean ± S [-]	RSD [%]	Mean ± S [-]	RSD [%]
1	1.000 ± 0.000	0.0	1.000 ± 0.000	0.0	1.000 ± 0.000	0.0	1.000 ± 0.000	0.0	1.000 ± 0.000	0.0	1.000 ± 0.000	0.0	1.000 ± 0.000	0.0
2	0.133 ± 0.008	5.8	0.111 ± 0.008	7.2	0.116 ± 0.004	3.7	0.104 ± 0.004	3.6	0.105 ± 0.006	5.4	0.096 ± 0.005	5.3	0.100 ± 0.003	3.5
3	0.433 ± 0.003	0.7	0.429 ± 0.002	0.6	0.418 ± 0.006	1.6	0.421 ± 0.010	2.3	0.415 ± 0.002	0.4	0.411 ± 0.007	1.7	0.399 ± 0.005	1.2
4	0.261 ± 0.007	2.6	0.250 ± 0.001	0.6	0.242 ± 0.002	0.8	0.249 ± 0.006	2.6	0.243 ± 0.006	2.6	0.242 ± 0.006	2.5	0.235 ± 0.004	1.5
5	0.104 ± 0.001	1.1	0.105 ± 0.004	3.5	0.103 ± 0.005	4.7	0.102 ± 0.004	3.5	0.103 ± 0.002	1.6	0.099 ± 0.003	3.4	0.102 ± 0.002	2.4
6	0.181 ± 0.002	0.9	0.179 ± 0.007	3.7	0.183 ± 0.002	1.2	0.174 ± 0.003	1.9	0.183 ± 0.002	0.9	0.171 ± 0.002	1.4	0.176 ± 0.003	1.6
7	0.059 ± 0.002	2.7	0.061 ± 0.003	4.4	0.057 ± 0.004	6.5	0.056 ± 0.005	8.9	0.057 ± 0.003	5.6	0.056 ± 0.005	8.6	0.056 ± 0.005	9.4
8	0.137 ± 0.004	3.0	0.146 ± 0.010	6.6	0.149 ± 0.003	1.8	0.144 ± 0.003	1.9	0.159 ± 0.004	2.3	0.141 ± 0.003	2.3	0.166 ± 0.003	1.6

Table S2. (**b**) Octane

Sensor No.	Month 1		Month 7		Month 13		Month 19		Month 25		Month 31		Month 37	
	Mean ± S [-]	RSD [%]	Mean ± S [-]	RSD [%]	Mean ± S [-]	RSD [%]	Mean ± S [-]	RSD [%]	Mean ± S [-]	RSD [%]	Mean ± S [-]	RSD [%]	Mean ± S [-]	RSD [%]
1	n/a	n/a	1.000 ± 0.000	0.0	1.000 ± 0.000	0.0	1.000 ± 0.000	0.0	1.000 ± 0.000	0.0	1.000 ± 0.000	0.0	1.000 ± 0.000	0.0
2	n/a	n/a	0.102 ± 0.011	10.7	0.097 ± 0.001	1.2	0.093 ± 0.002	2.6	0.092 ± 0.002	2.6	0.087 ± 0.004	4.8	0.090 ± 0.002	2.4
3	n/a	n/a	0.222 ± 0.018	7.9	0.212 ± 0.015	7.3	0.220 ± 0.014	6.5	0.220 ± 0.013	5.9	0.215 ± 0.012	5.4	0.213 ± 0.012	5.5
4	n/a	n/a	0.087 ± 0.004	5.0	0.086 ± 0.003	3.6	0.085 ± 0.003	3.5	0.088 ± 0.003	3.6	0.085 ± 0.001	1.7	0.087 ± 0.002	2.5
5	n/a	n/a	0.184 ± 0.010	5.3	0.165 ± 0.002	1.0	0.164 ± 0.002	1.0	0.164 ± 0.002	0.9	0.157 ± 0.005	3.1	0.159 ± 0.003	1.7
6	n/a	n/a	0.415 ± 0.016	3.8	0.404 ± 0.007	1.6	0.397 ± 0.006	1.4	0.413 ± 0.006	1.6	0.383 ± 0.001	0.3	0.392 ± 0.008	2.0
7	n/a	n/a	0.112 ± 0.006	5.4	0.107 ± 0.003	2.8	0.113 ± 0.002	1.6	0.116 ± 0.001	1.3	0.117 ± 0.001	0.9	0.119 ± 0.001	0.9
8	n/a	n/a	0.693 ± 0.047	6.8	0.685 ± 0.026	3.8	0.691 ± 0.027	3.9	0.710 ± 0.031	4.3	0.699 ± 0.028	4.0	0.714 ± 0.024	3.3

Table S2. (**c**) Xylene

Sen-sor No.	Month 1		Month 7		Month 13		Month 19		Month 25		Month 31		Month 37	
	Mean ± S [-]	RSD [%]	Mean ± S [-]	RSD [%]	Mean ± S [-]	RSD [%]	Mean ± S [-]	RSD [%]	Mean ± S [-]	RSD [%]	Mean ± S [-]	RSD [%]	Mean ± S [-]	RSD [%]
1	1.000 ± 0.000	0.0	1.000 ± 0.000	0.0	1.000 ± 0.000	0.0	1.000 ± 0.000	0.0	1.000 ± 0.000	0.0	1.000 ± 0.000	0.0	1.000 ± 0.000	0.0
2	0.103 ± 0.006	6.1	0.086 ± 0.009	9.9	0.089 ± 0.006	6.7	0.078 ± 0.003	3.5	0.078 ± 0.002	1.9	0.072 ± 0.002	3.4	0.076 ± 0.003	4.2
3	0.586 ± 0.028	4.7	0.586 ± 0.025	4.3	0.541 ± 0.035	6.5	0.567 ± 0.010	1.8	0.535 ± 0.045	8.5	0.547 ± 0.011	2.0	0.509 ± 0.027	5.2
4	0.169 ± 0.005	2.7	0.151 ± 0.005	3.4	0.147 ± 0.007	5.0	0.150 ± 0.003	1.7	0.141 ± 0.003	2.0	0.145 ± 0.003	1.9	0.141 ± 0.003	1.8
5	0.148 ± 0.003	1.9	0.137 ± 0.005	3.3	0.132 ± 0.002	1.7	0.129 ± 0.004	3.3	0.133 ± 0.005	4.0	0.125 ± 0.005	4.1	0.127 ± 0.002	1.7
6	0.257 ± 0.002	0.8	0.249 ± 0.003	1.2	0.254 ± 0.003	1.1	0.244 ± 0.003	1.1	0.255 ± 0.002	0.8	0.232 ± 0.006	2.6	0.240 ± 0.004	1.7
7	0.065 ± 0.001	1.4	0.072 ± 0.004	5.5	0.068 ± 0.001	1.8	0.070 ± 0.001	1.7	0.072 ± 0.002	2.7	0.072 ± 0.001	1.6	0.073 ± 0.001	1.1
8	0.257 ± 0.018	7.1	0.272 ± 0.013	4.7	0.295 ± 0.019	6.5	0.282 ± 0.021	7.3	0.332 ± 0.018	5.4	0.285 ± 0.032	11.3	0.347 ± 0.010	2.8
